# Web-based interventions to promote physical activity by older adults: promising perspectives for a public health challenge

**DOI:** 10.1186/0778-7367-71-16

**Published:** 2013-07-02

**Authors:** Alexandre Mouton, Marc Cloes

**Affiliations:** 1 Department of Sports and Rehabilitation Sciences, University of Liège, Allée des Sports, 4, 4000, Liège, Belgium

**Keywords:** Physical activity, Web-based intervention, Older adults, Exercise, Internet, Technology, Computer, Physical activity promotion

## Abstract

Regular physical activity is associated with a wide range of health benefits. As population age, promotion of physical activity should specifically target older adults, an expanding group involving potential higher health care costs in the near future. Innovative interventions focusing on physical activity behaviors of senior adults exposed promising results, most recently through the use of the Internet. If seniors and Internet are generally considered as two opposite concepts, arguments in favour of bringing them together in a public health perspective have been identified by the recent literature. Older adults are the fastest growing group of Internet users and are more prone than younger to use it for health-related subjects. Web-based interventions are effective in many health promotion sectors, including physical activity. This is particularly true when interventions target the environmental determinants of each senior citizen and are specifically designed for this population. Those early research findings must clearly be extended, particularly regarding to the long term effects of Web-based physical activity interventions. Solutions that will reduce the high dropout rate recorded in the existing literature must also be considered as a priority in order to ensure the development of this forward-looking field of research.

## Background

If the numerous health benefits of physical activity (PA) could be gathered into a pill, this would simply be the most prescribed drug in the world [1]. This statement is clearly appropriate since regular PA dramatically reduces the risk of chronic diseases such as cardiovascular diseases, diabetes, osteoporosis, several types of cancer and even depression [2]. As people age, PA offers great opportunities to increase active and independent life expectancies [3,4]. Beyond life expectancy, healthy life years could be increased by 2020 in the EU by 2.0 years for man and 1.4 years for women [5] in setting up adequate preventive strategies for healthier lifestyles, such as PA. Despite these proven health benefits, population in Western countries meet less and less the public health recommendations for PA when they get older [6,7]. If seniors are the most growing age group and generate the highest health care costs [7], the development of effective interventions promoting PA among this population is dramatically needed.

Research has shown that interventions targeting PA behavior of older adults can be effective [8,9]. Innovative strategies to reach this large and emerging population could be considered as a critical health challenge. Recent studies have shown that Web-based interventions to promote PA exposed promising effects [10], with at least as many positive outcomes as non Web-based interventions, and at a lower cost [11,12]. Nevertheless, an uninformed public may be sceptical about implementing Web-based PA in older adults. The common popular belief arguing that seniors may be less prone than younger age groups to adopt new technological approach remains highly widespread. Then, one could address this question of major interest: “Are older adults ready for a Web-based PA promotion?” In this paper, we questioned the existing literature to provide a summarized and opinionated answer to this innovative issue. The low number of papers available in this specific field of research dissuades us to propose a systematic review that would have been not relevant at this time.

## Main text and discussion

### Seniors’ use of internet

Older adults are the fastest growing group of Internet users. In 2012, 58% of the Belgian older adults (55–74 years) were regularly connected to the web [13]. Furthermore, Internet use among this age group will increase in the near future because baby boomers aged 50 to 64 years are twice as likely as older adults (>64 years) to use the Internet to research information [14]. Moreover, older adults are more prone than younger people to use the Internet for health-related subjects [15]. As senior population is generally more concerned about health [16], we can consider that Web-based PA promotion research must actively explore how to maximize its impact, particularly to socioeconomically disadvantaged groups with restricted access to the digital technologies. Special attention should then be paid to the bridging of this digital divide.

### Effective in health promotion

Web-based interventions are already widely used in the health promotion sector (eHealth). If interventions focusing on dietary behavior [17], tobacco use [18], alcohol consumption [19] or depression [20] are already successfully proposed on the Internet, even with seniors [21], there is no reason for excluding PA from this innovative approach. Moreover, the ongoing development of empirically based guidelines for tailoring health behaviors on the Web [22] could be relevant for PA promotion.

### Effective for older adults

Research on Web-based PA promotion among older adults is still at an early stage of development. This computer technology was first proposed via an automated computer system [23], evolving to a computer-tailored [24], and finally to a Web-based format [25-28]. At each stage of this development, significant effects on PA behavior were found [23,24,26-28]. Those results are particularly of interest because benefits were also observed among sedentary people [27], and even among the elderly people [26]. If evidence about the long-term (one year) effectiveness of computer-tailored PA interventions for older adults is already available [29], literature is still lacking concerning long term effects of Web-based interventions. Positive impact of Web-based PA interventions primarily concerns self-reported PA level [24,26,27,29], or even perceived barriers to PA [28,29]. Those results underline that the existing literature about Web-based interventions to promote PA by older adults is exclusively assessed by self-reported measures. It should therefore be kept in mind that people often overreport their physical activity [30]. The use of objective measures to assess PA is therefore recommended for future research.

### Targeting environmental determinants

Environmentally tailored PA Websites could result in extra intervention effects on PA behaviors [24,31]. Indeed, a local neighborhood-environment focused PA website could be more effective at engaging participants than a motivational-information website [31]. Inclusion of database of local opportunities for PA, calendar of PA events in the community, maps of walking and cycling trails, or online forums providing support in finding a sport mate are then widely recommended. The use of state-of-the-art communication technologies in PA promotion is needed, especially as mobile devices are increasingly used, and even lead to improved PA levels among older adults [32]. Those up-to-date communication tools provide opportunities for the participants to exchange information about their direct PA environment, including GPS technology that gives them access to cultural and touristic information during their walks.

### Make it suitable for older adults

The development of Web-based PA interventions for older adults involves a twofold adaptation. First, the most effective interventions to promote PA must be adjusted to the specificity of an online environment [33]. Interventions delivered via the Internet have the advantage to provide an individualized and tailored interface, with materials adapted to the individual characteristics and interest. Accessible anytime and anywhere, this interface has also the potential to provide more interactive (e.g., PA forum) and multimedia (e.g., videos) applications in a theorically-driven format. However, the added values of those specific components and of a theory-based approach for a Web-based PA intervention still need to be clearly demonstrated [10]. The further adaptation concerns older adults. A first specific care is about including examples of exercises that senior participants could do safely on their own, at home or in their neighbouring environment, with minimum equipment. Moreover, particular attention should be paid to the design of the Website, which could lead to an increase of the PA level when the interface is more interactive [34]. Tools have also been developed to create more “senior friendly” Websites with several adaptations, such as a larger text size, a break of lengthy text into short sections, or a single mouse click to access to the information [35]. Finally, current literature is very scattered regarding the theoretical base used for the Web-based interventions to promote PA by older adults. Some studies use a protocol which combines several theoretical models [24-26,29] (i.e., the intervention mapping protocol [36]) while others are based on a unique model [27,28](i.e., the theory of planned behaviour [37]). The usage of a theory-based intervention may be an indicator of higher quality content and could be evaluated by a validated assessment template for the use of behaviour change theories in PA Websites [38]. This 118 items evaluation template, including five categories of interaction strategies (knowledge, cognitive strategies, behavioral strategies, emotion-focused strategies, and therapeutic interventions) at five levels of interaction with the user (information or guidelines, assessment, feedback, general assistance, and individually tailored assistance), could be extended to the specific target group of older adults for a more reliable assessment of future interventions.

### Reduce the high attrition rate

One of the most important failing of the Web-based PA promotion among older adults is about the high dropouts levels recorded in nearly all existing research protocols [25-27,31]. Approximately a 30% dropout rate is expected in a Web-based PA intervention, with systematically higher rates observed in the intervention groups compared to the control groups [27,29]. Limited skills in using the Internet and a subsequent lack of motivation could still represent a barrier to the participation of this age group. Computer training for senior adults should therefore be stimulated and incorporated in PA promotion interventions in order to increase their self-efficacy regarding the Internet tool. In its systematic review, Brouwer et al. [39] exposed that only peer support, counsellor support, email or phone contact with the participants and regular updates of the website resulted in longer visit and more logins on the Website. Those statements are particularly of interest to reach people with low intention to be physically active who are generally less motivated by health-promoting interventions. This group definitively needs additional motivators. It may be then necessary to make Websites more interactive, dynamic, and appealing for senior adults in order to extend their exposure and their engagement to a PA promotion program [40]. Developing effective Web-based interventions to stimulate PA participation among older people is then a promising challenge. Social and gaming elements, adapted for this specific group, are some examples of components that could make the intervention more exciting. More research is then clearly needed to explore strategies that limit the high attrition rate in Web-based PA promotion among older people.

## Conclusion

Web-based interventions designed to promote physical activity by older adults are still at an early stage of development. Nevertheless, research findings about Web-based interventions in other health promotion programs and among younger people are clearly encouraging. Table 1 provides a short overview of the current strengths, weaknesses, opportunities, and threats (SWOT) of the Web-based interventions to promote physical activity by older adults that has been pointed out in this article. The identification of the key internal (*Strengths* and *Weaknesses*) and external (*Opportunities* and *Threats*) factors are important to suggest recommendations for subsequent research in the field.

**Table 1:** Swot analysis of the web-based interventions to promote physical activity by older adults

	
**Strengths**	**Weaknesses**
Evidence for a short term effectiveness	Large amount of senior Internet non-users
Environmentally tailored interventions	Lack of social/interactive elements
Cost-effective interventions	High dropout rates
Seniors’ concerns about their health	Self-reported measures to assess PA
**Opportunities**	**Threats**
Create more “senior-friendly” Websites	Uncertain long-term effectiveness
Combine with social/cultural/gaming elements	Fail to reach seniors with low motivation for PA
Standardize theorically-driven Websites	Rise of the digital divide
Use objective measures to assess PA	

In light of Table 1, if we consider that the suitability of the Internet tool for seniors is still a preoccupying issue, this turns out to be a temporary one. The next generation of seniors is actually much more likely to use new online technologies. However, particular attention must be paid to provide solutions that will reduce the high dropout rate recorded in the existing literature. Usage of senior-friendly Websites with original motivating components and including technologies that connect the participant to its close environment, are some examples of groundbreaking strategies that research must carry on in the near future. New technologies and older adults, two apparently opposite fields, could therefore be joined together for the welfare of public health.

## Abbreviations

PA: Physical activity.

## Competing interests

The authors declare that they have no competing interests.

## Authors’ contribution

AM wrote the first draft of the manuscript. MC revised it critically and has given final approval of the version to be published. Both authors read and approved the final manuscript.

## Authors’ information

AM is a PhD student involved in the physical activity promotion field, especially among older people. This includes researches about Web-based interventions implemented at the community level. MC is a professor specialized in physical activity promotion at all levels and in the physical education field.
